# Impact of Scoring Balloon Angioplasty on Lesion Preparation for DCB Treatment of Coronary Lesions

**DOI:** 10.3390/jcm12196254

**Published:** 2023-09-28

**Authors:** Eun-Seok Shin, Soe Hee Ann, Mi Hee Jang, Bitna Kim, Tae-Hyun Kim, Chang-Bae Sohn, Byung Joo Choi

**Affiliations:** 1 Department of Cardiology, Ulsan University Hospital, University of Ulsan College of Medicine, Ulsan 44033, Republic of Korea; 0732568@uuh.ulsan.kr (S.H.A.); 0736353@uuh.ulsan.kr (M.H.J.); binna9044@gmail.com (B.K.); 2 Department of Cardiology, Ulsan Medical Center, Ulsan 44686, Republic of Korea; rozze33@gmail.com (T.-H.K.); scb77@daum.net (C.-B.S.); md65753@hanmail.net (B.J.C.)

**Keywords:** scoring balloon, drug-coated balloon, balloon angioplasty, dissection, coronary artery disease

## Abstract

Objective: The aim of this study was to evaluate the efficacy of scoring balloon angioplasty for drug-coated balloon (DCB) treatment in percutaneous coronary intervention. Background: The scoring balloon angioplasty may play a pivotal role in enhancing the outcomes of DCB treatment. Methods: A total of 259 patients (278 lesions) with coronary artery disease successfully treated with DCB were retrospectively enrolled. The mean age of the patients was 62.2 ± 11.1 years, and the majority of patients were men (68.7%). The study’s endpoint was defined as achieving an optimal angiographic result, which consisted of Thrombolysis in Myocardial Infarction (TIMI) flow grade 3, residual diameter stenosis ≤ 30%, and dissection less than type C after the procedure. Results: Angioplasty was performed for 61 lesions with a scoring balloon and 217 lesions with a non-scoring balloon. All lesions were TIMI flow grade 3 except two lesions in the non-scoring balloon group. The scoring balloon group had a higher prevalence of residual diameter stenosis ≤ 30% (68.9% vs. 39.6%, *p* < 0.001), while severe dissection, defined as type C or greater, was observed less frequently (9.8% vs. 31.8%, *p* = 0.001). Moreover, the scoring balloon group achieved a superior rate of optimal angiographic results (60.7% vs. 28.6%, *p* < 0.001). In multivariable analysis, scoring balloon (OR: 3.08 [95% confidence interval, 1.47–6.58], *p* = 0.003) and DCB balloon-to-artery ratios (OR: 5.46 [95% confidence interval, 1.43–21.93], *p* = 0.014) were independent factors in the increasing rate of optimal angiographic result. Conclusions: The application of a scoring balloon catheter for lesion preparation, aiming to make them suitable for DCB treatment, was associated with a decreased risk of severe dissection and a greater occurrence of optimal angiographic outcomes compared with non-scoring balloon angioplasty.

## 1. Introduction

For successful drug-coated balloon (DCB) treatment, optimal lesion preparation is an essential factor for percutaneous coronary intervention (PCI) in coronary artery disease (CAD). The importance of lesion preparation is emphasized by both the International DCB Consensus Group and the Asia-Pacific DCB Consensus Group [1,2]. They define an acceptable angiographic result as no flow-limiting dissections, residual diameter stenosis less than 30%, or an FFR value greater than 0.80 (≥0.75 in Asia-Pacific Consensus). In a previous study, it was shown that when the DCB-Consensus-recommended lesion preparation yielded an acceptable angiographic result, the clinical outcome was better than that of patients who did not [3]. They conclude that it is important to ensure the recommended criteria of the Consensus Group are fulfilled before choosing a DCB catheter.

In the process of preparing the lesion, balloon angioplasty is the basic technique, and currently, semi-compliant plain balloons are commonly used as the standard choice. However, several studies have indicated that using scoring balloon angioplasty enhances lumen gain [4] and improves the effectiveness of DCB therapy [5] resulting in a lower risk of target lesion failure [6] compared with the standard lesion preparation. In the ISAR-DESIRE 4 study, when drug-eluting stent (DES) restenosis lesions were treated with DCB, predilation using a scoring balloon and standard lesion preparation were randomly compared. The primary endpoint, in-segment diameter stenosis at 6 to 8 months, was lower in the scoring balloon group (35.0 ± 16.8% vs. 40.4 ± 21.4%, *p* = 0.047) and had less binary angiographic restenosis (18.5% vs. 32.0%, *p* = 0.026) [5]. While these studies have shown the effectiveness of scoring balloons in DCB treatment for stent restenosis, there are limited data on the use of scoring balloon angioplasty for DCB treatment of coronary lesions. Therefore, the aim of this study was to investigate the impact of a scoring balloon on angiographic and clinical outcomes in the treatment of CAD with DCB.

## 2. Methods

### 2.1. Study Population

A total of 259 patients (278 lesions) who underwent successful PCI for CAD using DCB alone were retrospectively included in this study. The data were collected from October 2018 to November 2020 at two teaching hospitals in South Korea (Ulsan University Hospital, Ulsan Medical Center), both of which had prior experience in treating patients with CAD using DCB (“Impact of Drug-Coated Balloon Treatment in de Novo Coronary Lesion”; NCT04619277). The mean age of the patients was 62.2 ± 11.1 years, and the majority of patients were men. Exclusion criteria were previously undergone coronary artery bypass surgery, severe left ventricular dysfunction (ejection fraction < 35%), chronic kidney disease, ST-segment elevation myocardial infarction requiring primary PCI, heavily calcified or thrombotic lesion, failed PCI for target lesions, and a life expectancy of <1 year. The study protocol was approved by the institutional review board of each participating center, and all patients provided written informed consent at the time of enrollment.

### 2.2. Target Lesion Preparation and DCB Treatment

All patients were pretreated with aspirin 200 mg and clopidogrel 300 or 600 mg as loading doses, followed by intravenous injection of 100 U/kg unfractionated heparin to maintain an activated clotting time of ≥250 s during the procedure. Intracoronary nitroglycerin (200 µg) was administered before diagnostic coronary angiography was performed. For the DCB treatment, the intervention was performed according to international and Asia-Pacific consensus recommendations [1,2]. Firstly, the target lesion was dilated using an optimal-sized balloon, with a balloon-to-artery ratio of 0.8–1.0, as determined by angiography [1,2]. If the optimal-sized balloon failed to reach the lesion, a smaller-sized balloon was used for lesion preparation, followed by the use of the optimal-sized balloon. After that, the decision of whether to use scoring balloons or non-scoring balloons for lesion preparation rested with the operator. For scoring balloons, 70.5% of cases used AngioSculpt™ catheter (Philips Healthcare, Cambridge, MA, USA) and 29.5% used Lacrosse non-slip element NSE Alpha™ (Goodman Co, Ltd., Nagoya, Japan). For non-scoring balloons, semi-compliant balloons were used in most cases (97.7%), and non-compliant balloons were used in 2.3%. For DCB treatment, a SeQuent Please™ was used (B. Braun, Melsungen, Germany), which was delivered to the target lesion and inflated with nominal pressure for a duration of 60 s.

### 2.3. Angiographic Measurement and Study Endpoint

Angiography was performed after the administration of 200 µg of intracoronary nitroglycerine in at least two orthogonal projections before and after the procedure. Quantitative analysis of angiographic data was performed offline by a single independent expert in blinded core lab (Cardiovascular Research Foundation in Dong-A University Hospital) using the validated software (CAAS II, Pie Medical Imaging). The following parameters were analyzed: reference vessel diameter, minimal lumen diameter (MLD), percent diameter stenosis, lumen gain (defined as the value obtained by subtracting MLD after procedure from MLD before procedure), and lesion length. Measurements included the whole segment treated plus 5 mm proximally and distally. In the post-lesion preparation angiography, coronary dissection was assessed and graded from A to F (with A being the lowest grade and F the highest grade) according to the National Heart, Lung, and Blood Institute (NHLBI) classification [7].

The study endpoint was defined as achieving an optimal angiographic result, which consisted of Thrombolysis in Myocardial Infarction (TIMI) flow grade 3, residual diameter stenosis ≤ 30%, and dissection less than type C after the procedure.

### 2.4. Patient Follow-Up

All 259 patients underwent a clinical follow-up following the index procedure via telephone interviews and outpatient clinic visits. We conducted an analysis of cumulative major adverse cardiac events (MACE), which is a composite outcome comprising cardiac death, myocardial infarction, target lesion thrombosis, and target vessel revascularization at 1 year. Cardiac death was defined as any death that was not clearly of extracardiac origin, including myocardial infarction, according to previously published guidelines [8]. Additionally, probable or definite stent thrombosis was defined according to the Academic Research Consortium definition [9].

### 2.5. Statistical Analysis

Clinical characteristics are reported as percentages for categorical variables and means with standard deviations for continuous variables. Comparisons between groups were made using either the Pearson’s chi-squared test or the Fisher’s exact test for categorical variables and the Student’s *t*-test for continuous variables, as appropriate. Logistic regression model was used to calculate odds ratio (OR) and 95% CIs; logistic regression was used to examine associations between scoring balloon and optimal angiographic results. All *p*-values were two-sided, and a value of <0.05 was considered statistically significant. R version 4.1.2 (R Foundation for Statistical Computing, Vienna, Austria) was used for all statistical analyses in this study.

## 3. Results

### 3.1. Baseline Clinical and Procedure Characteristics

The baseline clinical and procedural characteristics of the patients are summarized in Table 1. The left anterior descending artery lesion was more frequently observed in the scoring balloon group, while the left circumflex artery lesion was more prevalent in the non-scoring balloon group. The SYNTAX score and total number of treated vessels were comparable in both groups. In the scoring balloon group, the DCB diameter was larger, and the inflation time was longer. However, there was no difference in the DCB balloon-to-artery ratios between the groups. Notably, severe dissections of type C or higher were significantly less frequent in the scoring balloon group compared with the non-scoring balloon group (9.8% vs. 31.8%, *p* = 0.001).

### 3.2. Angiographic Results and Study Endpoint

**Figure 1:**
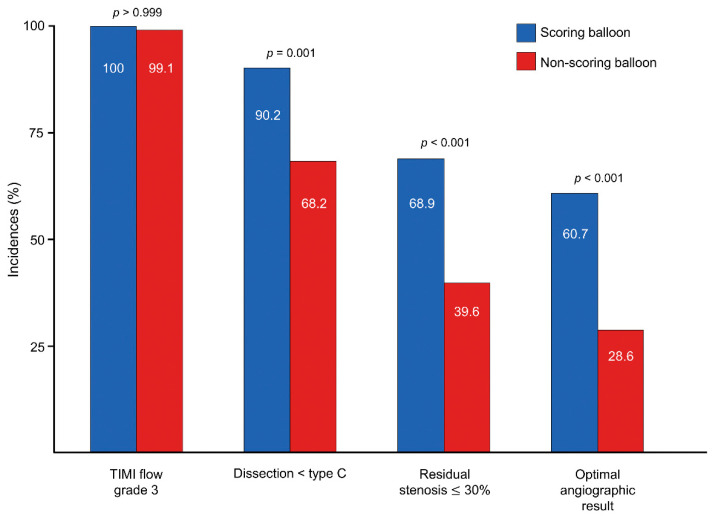
Incidences of optimal angiographic result in scoring balloon and non-scoring balloon groups.

The quantitative angiographic data of the lesions are summarized in Table 2. The baseline characteristics of the lesion were different between the two groups. The reference vessel diameter and MLD were larger in the scoring balloon group, while the lesion length and diameter stenosis were greater in the non-scoring balloon group. After the procedure, the scoring balloon group still had a larger MLD and a smaller diameter stenosis compared with the other group. Furthermore, the scoring balloon achieved a greater increase in lumen gain (1.10 ± 0.38 mm vs. 0.94 ± 0.42 mm, *p* = 0.009). They also had more optimal angiographic results, consisting of TIMI flow grade 3, residual stenosis of 30% or less, and dissection less than type C, compared with the non-scoring balloon group (60.7% vs. 28.6%, *p* < 0.001) (Figure 1). All lesions were TIMI flow grade 3 except two lesions in the non-scoring balloon group. The scoring balloon group had a higher prevalence of residual diameter stenosis ≤ 30% (68.9% vs. 39.6%, *p* < 0.001), while severe dissection, defined as type C or greater, was observed less frequently (9.8% vs. 31.8%, *p* = 0.001). When assessing lumen gain based on dissection severity, the scoring balloon group exhibited significantly greater lumen gain compared with the non-scoring balloon group in cases without dissection.

### 3.3. Clinical Outcomes

**Figure 2:**
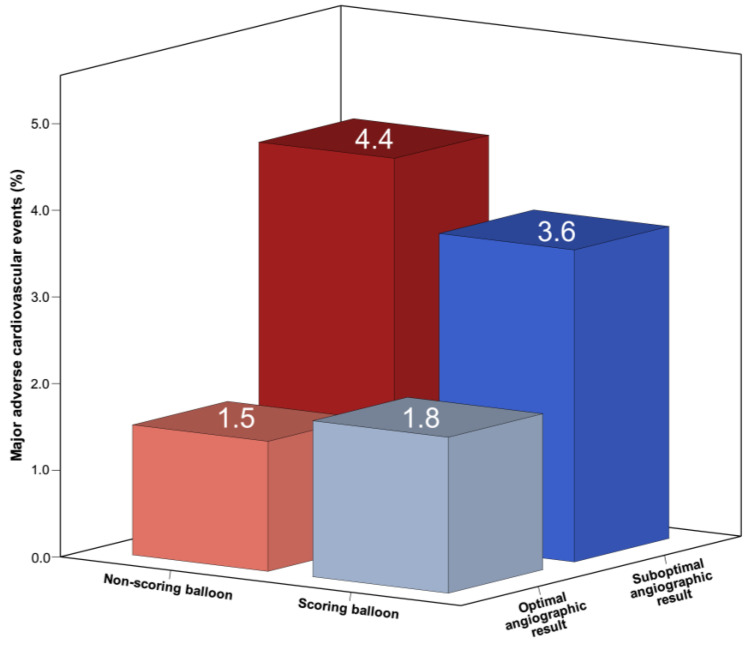
The major adverse cardiovascular events in scoring balloon and non-scoring balloon groups according to the presence of optimal angiographic result at 1-year follow-up.

The MACE consisting of cardiac death, myocardial infarction, target lesion thrombosis, and target vessel revascularization at 1 year occurred comparably in both groups (5.4% in the scoring balloon group vs. 5.9% in the non-scoring balloon group) (Table 3). Nevertheless, the presence of suboptimal angiographic results showed a tendency towards an increased occurrence of adverse events, particularly in cases where the non-scoring balloon group exhibited suboptimal angiographic outcomes (Figure 2). However, the limited number of events resulted in insufficient statistical power, precluding any definitive conclusions.

### 3.4. Independent Predictors Associated with Severe Dissection and Optimal Angiographic Result

In the multivariable analysis, the scoring balloon was identified as an independent predictor of severe dissection (odds ratio: 0.18 [95% confidence interval, 0.05–0.49], *p* = 0.002) (Table 4). Additionally, two independent factors associated with an increased rate of optimal angiographic results were the scoring balloon (odds ratio: 3.08 [95% confidence interval, 1.47–6.58], *p* = 0.003) and the DCB balloon-to-artery ratio (odds ratio: 5.46 [95% confidence interval, 1.43–21.93], *p* = 0.014).

## 4. Discussion

The main findings of this study are as follows. (1) The scoring balloon angioplasty demonstrated a significantly greater increase in lumen gain. (2) Severe dissection, defined as type C or greater, was less frequently observed in the scoring balloon group. (3) The scoring balloon group achieved a higher rate of optimal angiographic results compared with the non-scoring balloon group. (4) Suboptimal angiographic results were associated with a tendency towards a higher incidence of adverse events, especially when the non-scoring balloon group had suboptimal angiographic outcomes.

Despite the crucial significance of appropriate lesion preparation for the successful treatment of DCB in coronary lesions, there is a scarcity of available data on this matter. In particular, the data are limited on the impact of scoring balloon angioplasty on coronary lesions. The attainment of adequate luminal gain before performing DCB treatment assumes significant importance in cases where stent or scaffold implantations are not performed. In the follow-up angiogram after plain balloon angioplasty, late lumen loss occurs three times more and restenosis occurs in about one-third of lesions compared with after DCB treatment [10]. However, as shown in several studies, after DCB treatment, the late lumen loss appears to be about 0.05 mm at 6- to 9-month follow-up, suggesting that the lumen gain post procedure is maintained [11,12,13,14]. In addition, in more than half, late lumen enlargement occurs, resulting in a larger lumen than post procedure [15]. These findings highlight the significance of achieving sufficient lumen gain through proper lesion preparation before DCB treatment. Additionally, in this study, the scoring balloon group demonstrated a higher acute lumen gain compared with the non-scoring balloon group.

Balloon angioplasty serves as a fundamental technique, with semi-compliant plain balloons commonly used as the standard option in current practice. However, in an effort to streamline the procedure and improve stent expansion, specialized scoring balloons were developed for predilating complex lesions. A previous study demonstrated that pretreatment with the AngioSculpt balloon improved stent expansion and reduced the disparity between predicted and achieved stent dimensions [16]. Even in cases of in-stent restenosis lesions, the use of scoring balloons has shown potential in enhancing neointima modification and improving the efficacy of DCB therapy. Comprehensive predilation ensures optimal surface contact between the DCB and the underlying neointimal plaque, facilitating effective drug transfer. Furthermore, preclinical data indicate that moderate localized injury may enhance the delivery and tissue retention of the antirestenotic agent [17,18]. In the ISAR-DESIRE 4 trial, scoring balloon predilation and standard lesion preparation were compared for plaque modification in DES restenosis before DCB treatment. The results revealed that using the scoring balloon for neointimal modification before DCB angioplasty led to superior angiographic outcomes during follow-up. While no significant difference in the overall outcome was observed, both treatment approaches exhibited a high level of clinical safety with comparable low event rates for up to 1 year. However, the primary endpoint, which measured the in-segment percentage diameter stenosis in 6- to 8-month follow-up angiography, was found to be lower in the scoring balloon group (35.0 ± 16.8% vs. 40.4 ± 21.4%, *p* = 0.047). Several studies have also shown that the application of scoring balloon angioplasty results in improved stent expansion, consequently reducing the risk of target lesion revascularization and target vessel revascularization compared with conventional lesion preparation methods [4,16]. Although studies investigating the impact of scoring balloons on coronary lesions are limited, a recent study demonstrated that the systematic application of scoring balloon catheters for preparing lesions suitable for DCB treatment is linked to a high rate of procedural success and a low occurrence of target lesion failure (including rates for target lesion revascularization, myocardial infarction, or cardiac death at 9 months) in coronary lesions [6]. However, given the non-randomized and observational nature of this study, a well-designed randomized controlled trial is essential in the future to validate the lesion-preparation effect of scoring balloons for successful DCB treatment.

## 5. Study Limitations

First, this study had a fundamental limitation because it was observational in nature and relied on registry data. Additionally, allowing physicians to choose the treatment strategy, including the selection of predilation balloon devices, introduces the potential for selection bias. While scoring balloons are typically designed for treating complex coronary artery diseases, especially for angioplasty of calcified plaques, lesion preparation was consistently employed prior to DCB treatment, making it a routine practice. Consequently, the selection of the scoring balloon depended more on the operator’s preference than on the characteristics of the lesion. Upon examining the SYNTAX score, no significant differences were observed between the two groups. Second, the study population came from an expert center in DCB treatment for CAD. Thus, these results may not be reproducible without an adequate learning curve. To determine whether scoring balloon angioplasty is effective for treating CAD with DCB, it is crucial to conduct well-planned and large-scale randomized trials involving multiple specialized centers experienced in DCB treatment.

## 6. Conclusions

In this research, the utilization of a scoring balloon catheter aimed at preparing lesions for DCB treatment was found to be associated with a reduced risk of severe dissection and a higher rate of achieving optimal angiographic outcomes when compared with non-scoring balloon angioplasty. To further confirm the safety and effectiveness of the scoring balloon in DCB treatment, conducting larger randomized controlled trials in the future will be crucial.

## Figures and Tables

**Table 1:** Baseline clinical and procedure characteristics.

	Scoring Balloon (N = 56 Patients)	Non-Scoring Balloon (N = 203 Patients)	*p*-Value
Age, years	60.8 ± 9.1	62.7 ± 11.3	0.202
Men	40 (71.4)	138 (68.0)	0.741
Cardiovascular risk factors			
Hypertension	37 (66.1)	143 (70.4)	0.642
Diabetes	14 (25.0)	72 (35.5)	0.284
Dyslipidemia	47 (83.9)	156 (76.8)	0.461
Current smoking	13 (23.2)	50 (26.5)	0.352
Previous PCI	7 (12.5)	44 (21.7)	0.205
Clinical manifestations			0.573
Stable angina	21 (37.5)	67 (33.0)	
Unstable angina	22 (39.3)	90 (44.3)	
Non-ST-segment elevation myocardial infarction	11 (19.6)	33 (16.3)	
ST-segment elevation myocardial infarction	2 (3.6)	13 (6.4)	
Treated vessel	N = 61 vessels	N = 217 vessels	<0.001
Left main	2 (3.3)	1 (0.5)	
Left anterior descending	33 (54.1)	68 (31.3)	
Left circumflex	12 (19.7)	105 (48.4)	
Right coronary	14 (23.0)	43 (19.8)	
SYNTAX score	9.8 ± 5.5	10.3 ± 7.0	0.504
Total number of treated vessels	1.0 ± 0.2	1.0 ± 0.1	0.234
DCB treatment			
DCB diameter, mm	2.8 ± 0.4	2.5 ± 0.3	<0.001
DCB length, mm	23.6 ± 5.0	22.0 ± 5.2	0.041
Maximal inflation pressure, atm	9.6 ± 2.1	9.2 ± 2.3	0.259
Inflation time	67.7 ± 21.0	52.4 ± 18.3	<0.001
DCB balloon-to-artery ratio	1.0 ± 0.1	1.1 ± 0.2	0.337
Dissection type after procedure			
None	17 (27.9)	37 (17.1)	0.088
A	19 (31.1)	46 (21.2)	0.147
B	19 (31.1)	65 (30.0)	0.983
C	6 (9.8)	69 (31.8)	0.001

Values are presented as n (%) and mean ± standard deviation. Abbreviations: PCI = percutaneous coronary intervention; DCB = drug-coated balloon.

**Table 2:** Quantitative coronary angiography data.

	Scoring Balloon (N = 61 Vessels)	Non-Scoring Balloon (N = 217 Vessels)	*p*-Value
Before procedure			
Lesion length, mm	14.61± 6.36	17.41 ± 5.58	0.001
Reference vessel diameter, mm	2.74 ± 0.49	2.46 ± 0.48	<0.001
Minimum lumen diameter, mm	0.99 ± 0.35	0.71 ± 0.39	<0.001
Diameter stenosis, %	61.33 ± 11.47	70.79 ± 13.70	<0.001
After procedure			
Minimum lumen diameter, mm	2.08 ± 0.42	1.64 ± 0.43	<0.001
Diameter stenosis, %	26.29 ± 9.84	34.50 ± 11.93	<0.001
Lumen gain, mm	1.10 ± 0.38	0.94 ± 0.42	0.009
TIMI flow grade 3	61 (100)	215 (99.1)	>0.999
Residual stenosis ≤ 30%	42 (68.9)	86 (39.6)	<0.001
Dissection < type C	55 (90.2)	148 (68.2)	0.001
Optimal angiographic result	37 (60.7)	62 (28.6)	<0.001
Lumen gain according to dissection severity, mm			
None	1.31 ± 0.47	0.89 ± 0.42	0.002
A	0.96 ± 0.28	0.95 ± 0.43	0.969
B	1.05 ± 0.35	0.99 ± 0.42	0.601
C	1.10 ± 0.24	0.91 ± 0.43	0.273

Values are presented as n (%) and mean ± standard deviation. The optimal angiographic result consisted of TIMI flow grade 3, residual stenosis ≤ 30%, and dissection less than type C. Abbreviations: TIMI flow grade = Thrombolysis in Myocardial Infarction flow grade.

**Table 3:** Clinical events at 12-month follow-up according to the presence of an optimal angiographic result.

	Scoring Balloon (N = 56)		Non-Scoring Balloon (N = 203)	
	Optimal Angiographic Result (N = 34)	Suboptimal Angiographic Result (N = 22)	Optimal Angiographic Result (N = 55)	Suboptimal Angiographic Result (N = 148)
Major adverse cardiovascular events	1 (1.8)	2 (3.6)	3 (1.5)	9 (4.4)
Cardiac death	0	0	0	2 (1.0)
Myocardial infarction	0	0	0	0
Target lesion thrombosis	0	0	0	0
Target vessel revascularization	1 (1.8)	2 (3.6)	5 (1.5)	8 (3.9)

Values are presented as n (%). In four groups, the *p*-value was not significant above 0.05. Major adverse cardiovascular events were composed of cardiac death, myocardial infarction, target lesion thrombosis, and target vessel revascularization. The optimal angiographic result consisted of TIMI flow grade 3, residual stenosis ≤ 30%, and dissection less than type C.

**Table 4:** Multivariable analysis of factors associated with severe dissection and optimal angiographic result.

Variable	Severe Dissection		Optimal Angiographic Result	
	Odds Ratio (95% CI)	*p*-Value	Odds Ratio (95% CI)	*p*-Value
Age	0.99 (0.96–1.02)	0.359	1.00 (0.97–1.03)	0.995
Women	1.62 (0.82–3.20)	0.162	0.70 (0.35–1.35)	0.291
Hypertension	0.65 (0.33–1.30)	0.220	1.07 (0.55–2.13)	0.845
Diabetes	1.25 (0.64–2.44)	0.508	0.77 (0.39–1.50)	0.454
Dyslipidemia	1.03 (0.52–2.04)	0.936	1.00 (0.51–1.96)	0.996
Current smoking	0.73 (0.32–1.58)	0.433	1.14 (0.52–2.46)	0.731
Acute coronary syndrome	0.96 (0.50–1.91)	0.917	1.01 (0.53–1.96)	0.968
Left anterior descending artery	0.59 (0.29–1.15)	0.130	1.52 (0.82–2.81)	0.181
DCB balloon-to-artery ratio	0.69 (0.12–3.87)	0.679	5.46 (1.43–21.93)	0.014
DCB inflation time	1.01 (0.99–1.03)	0.380	1.00 (0.99–1.02)	0.574
Baseline diameter stenosis	0.99 (0.97–1.02)	0.566	0.99 (0.96–1.01)	0.310
Scoring balloon	0.18 (0.05–0.49)	0.002	3.08 (1.47–6.58)	0.003

The optimal angiographic result consisted of TIMI flow grade 3, residual stenosis ≤ 30%, and dissection less than type C. Abbreviations: DCB = drug-coated balloon; CI = confidence interval.

## Data Availability

The data presented in this study are available on request from the corresponding author.
